# Bioinformatic investigation of discordant sequence data for SARS-CoV-2: insights for robust genomic analysis during pandemic surveillance

**DOI:** 10.1099/mgen.0.001146

**Published:** 2023-11-29

**Authors:** Sara E. Zufan, Katherine A. Lau, Angela Donald, Tuyet Hoang, Charles S. P. Foster, Chisha Sikazwe, Torsten Theis, William D. Rawlinson, Susan A. Ballard, Timothy P. Stinear, Benjamin P. Howden, Amy V. Jennison, Torsten Seemann

**Affiliations:** ^1^​ Department of Microbiology and Immunology, The University of Melbourne at the Peter Doherty Institute for Infection and Immunity, Melbourne, VIC, Australia; ^2^​ RCPAQAP Biosecurity, St. Leonards, NSW, Australia; ^3^​ Microbiological Diagnostic Unit Public Health Laboratory, The University of Melbourne, at the Peter Doherty Institute for Infection and Immunity, Melbourne, Victoria, Australia; ^4^​ Serology and Virology Division (SAViD) SEALS Microbiology, NSW Health Pathology, Sydney, NSW, Australia; ^5^​ School of Biomedical Sciences, Faculty of Medicine and Health, University of New South Wales, Sydney, NSW, Australia; ^6^​ Department of Microbiology, PathWest Laboratory Medicine Western Australia, Nedlands, WA, Australia; ^7^​ School of Biomedical Sciences, The University of Western Australia, Nedlands, WA, Australia; ^8^​ School of Women’s and Children’s Health, Faculty of Medicine and Health, University of New South Wales, Sydney, NSW, Australia; ^9^​ School of Biotechnology and Biomolecular Sciences, Faculty of Science, University of New South Wales, Sydney, NSW, Australia; ^10^​ Public Health Microbiology, Forensic and Scientific Services, Queensland Department of Health, Brisbane, Australia

**Keywords:** bioinformatics, public health genomics, quality assurance, SARS-CoV-2

## Abstract

The COVID-19 pandemic has necessitated the rapid development and implementation of whole-genome sequencing (WGS) and bioinformatic methods for managing the pandemic. However, variability in methods and capabilities between laboratories has posed challenges in ensuring data accuracy. A national working group comprising 18 laboratory scientists and bioinformaticians from Australia and New Zealand was formed to improve data concordance across public health laboratories (PHLs). One effort, presented in this study, sought to understand the impact of the methodology on consensus genome concordance and interpretation. SARS-CoV-2 WGS proficiency testing programme (PTP) data were retrospectively obtained from the 2021 Royal College of Pathologists of Australasia Quality Assurance Programmes (RCPAQAP), which included 11 participating Australian laboratories. The submitted consensus genomes and reads from eight contrived specimens were investigated, focusing on discordant sequence data and findings were presented to the working group to inform best practices. Despite using a variety of laboratory and bioinformatic methods for SARS-CoV-2 WGS, participants largely produced concordant genomes. Two participants returned five discordant sites in a high-Cτ replicate, which could be resolved with reasonable bioinformatic quality thresholds. We noted ten discrepancies in genome assessment that arose from nucleotide heterogeneity at three different sites in three cell-culture-derived control specimens. While these sites were ultimately accurate after considering the participants’ bioinformatic parameters, it presented an interesting challenge for developing standards to account for intrahost single nucleotide variation (iSNV). Observed differences had little to no impact on key surveillance metrics, lineage assignment and phylogenetic clustering, while genome coverage <90 % affected both. We recommend PHLs bioinformatically generate two consensus genomes with and without ambiguity thresholds for quality control and downstream analysis, respectively, and adhere to a minimum 90 % genome coverage threshold for inclusion in surveillance interpretations. We also suggest additional PTP assessment criteria, including primer efficiency, detection of iSNVs and minimum genome coverage of 90 %. This study underscores the importance of multidisciplinary national working groups in informing guidelines in real time for bioinformatic quality acceptance criteria. It demonstrates the potential for enhancing public health responses through improved data concordance and quality control in SARS-CoV-2 genomic analysis during pandemic surveillance.

## Data Summary

The authors confirm that all supporting data, code and protocols have been provided within the article or through supplementary data files.

### Impact Statement

Amidst the COVID-19 pandemic, a unique collaboration between a national multidisciplinary working group and a quality assurance programme facilitated ongoing development of standardized quality control criteria and analysis methods for high-quality SARS-CoV-2 genomic approaches across Australia. With this article, we shed light on the robustness of amplicon sequencing and analysis methods to produce highly concordant genomes, while also presenting additional assessment criteria to guide laboratories in identifying areas for improvement. Insights from this nationwide collaboration underscore the need for real-time knowledge-sharing and iterative refinements to quality standards, particularly as situations and methods evolve during a pandemic. While the spotlight is on SARS-CoV-2, the analyses and findings have universal implications for genomic surveillance during infectious disease outbreaks. As WGS becomes increasingly central in outbreak surveillance, continuous evaluation and collaboration, like that described here, are vital to ensure data accuracy and inform future public health responses.

## Introduction

The global scientific community responded to the coronavirus disease (COVID-19) pandemic with the rapid development of laboratory and bioinformatic data analysis methods for whole-genome sequencing (WGS) of severe acute respiratory syndrome coronavirus 2 (SARS-CoV-2). These methods have been widely adopted, with more than 45 countries conducting routine genomic surveillance [1]. Genomic data have been used to inform the local and national pandemic response. Applications range from monitoring the distribution and emergence of variants of concern (VOC) to supplementing contact tracing efforts [2]. These efforts have been supported by an open-access framework for the wide-scale sharing of data and resources, including Global Initiative on Sharing All Influenza Data (GISAID), Nextstrain, and protocols.io, among others. By May 2020, problematic sequence data attributed to contamination or sequencing and bioinformatic errors had been detected in the public domain, highlighting the need for quality control and remediation strategies to improve consensus genome accuracy [3].

Amplicon sequencing is the main method of SARS-CoV-2 WGS, with bioinformatic methods dependent on the platform [4, 5]. Previous interlaboratory and interprotocol comparative studies of SARS-CoV-2 WGS have found differences in single nucleotide variants (SNVs) during the assessment of wet and dry methodologies [6, 7]. The appropriate selection of bioinformatic workflows and parameter thresholds is key to the accuracy of genomic data and varies depending on the sequencing approach and technology. While selection varies, some commonly accepted thresholds include the following: read depth (>=10 for Illumina, >=20 for Nanopore), phred score (>25) and genome coverage>=90 % [8]. Discrepancies at SARS-CoV-2 mutation sites can affect the interpretation of genomic metrics of clinical and public health importance, such as PANGO lineage classification, phylogenetic placement or genomic epidemiological clustering [9–11]. As such, bioinformatic quality control processes ensure that methodological variability yields consistent results.

Australia and New Zealand have been at the forefront of using fine-scale genomic data to manage their COVID-19 responses. Genomic data are shared via AusTrakka to provide context for local and interstate transmission. AusTrakka is a national real-time pathogen genomics surveillance platform for public health laboratories that facilitates analytical harmonization and national reporting as well as provides equitable access to computational resources and expertise [12]. Australia’s high case sequencing rate, combined with multijurisdictional data sharing, resulted in genomic epidemiological interpretations with as little as one SNV. As is common in many countries, each jurisdiction has a unique laboratory and bioinformatic workflow to suit its facilities.

In July 2020, the Medical Research Future Fund (MRFF) COVID-19 Quality Control Working Group (WG), supported by the Communicable Diseases Genomics Network (CDGN), initiated a concertorming guidelines in real timed effort to harness genomic sequencing for an enhanced response to the COVID-19 pandemic. Comprised of 18 public health laboratorians and bioinformaticians from Australia, the WG’s primary objective was to establish a framework that ensures laboratory and bioinformatic standards and quality control, permitting procedural adaptability yet ensuring consistent SARS-CoV-2 genomic data. Continuous communication among laboratory technicians and bioinformaticians was maintained via monthly meetings and regular online discussions via Slack, facilitating protocol optimization and addressing potential sequencing and analysis challenges in real time.

The WG aimed to enhance interlaboratory data concordance but faced challenges in coordinating a national validation study during pandemic surges. Consequently, the WG procured SARS-CoV-2 WGS proficiency testing programme (PTP) data from the Royal College of Pathologists of Australasia Quality Assurance Programmes (RCPAQAP) for retrospective, in-depth bioinformatic analyses. In the study presented here, PTP data were used to characterize discordant SNVs and investigate bioinformatic quality control standards for remediation. Submitted consensus genomes were used to evaluate the effect of discordant SNVs on key surveillance metrics, lineage assignment and phylogenetic clustering, while submitted read data were used to investigate discordant sites. Finally, we reflect on the importance of multidisciplinary national working groups in informing guidelines in real time for bioinformatic quality acceptance criteria under the competing demands of a pandemic.

## Methods

### Data Source: 2021 RCPAQAP SARS-CoV-2 WGS PTP

Data were obtained from the 2021 RCPAQAP SARS-CoV-2 WGS PTP, sample characteristics and methods have previously been described [13]. Briefly, viral RNA extracted from five unique isolates of SARS-CoV-2 were each diluted in 0.5 ml of nuclease-free water to assess the participant’s ability to sequence different variants and viral litres.

Contrived samples (*N*=8) were sent to 11 participating laboratories for WGS using each laboratory’s standard procedure. The raw reads of sequence data without preprocessing or trimming and the derived consensus sequence data, as submitted by the participants, were assessed based on three quality metrics (>50 % genome coverage, lineage assignment and >95 % accuracy), as decided with input from CDGN Genomics Implementation and Bioinformatics Working Groups (herein, *WG*) and previously described by Lau *et al*. [13]. The participants also completed a questionnaire documenting laboratory and bioinformatic methods performed and self-reported genomic characteristics.

Deidentified data were provided to the WG for the principal aim of defining QC criteria and data analysis methods for standardizing high-quality SARS-CoV-2 WGS, independent of the PTP. One participant belonging to the WG inadvertently omitted consensus genomes from their submission to the PTP and provided them voluntarily for this study. In this study, we refer to the participants by their laboratory number (LB01–LB11) and the samples by their biological specimen number (BS01–BS08) (Table 1).

**Table 1.** Characteristics of contrived specimen prepared for the PTP

Sample ID	Isolate (GISAID ID)	Average Cτ (E-gene NAT)	Lineage
BS01	EPI_ISL_406844	16.77	B
BS02	EPI_ISL_419750	16.95	B.1
BS07		24.14	
BS08		27.48	
BS03	EPI_ISL_480701	17.28	B.1.1.136
BS04	EPI_ISL_519314	17.31	D.2
BS05	EPI_ISL_563416	17.29	D.2
BS06	Negative control	Not detected	na

### Evaluation of consensus genomes

For the assessment of consensus genome quality, genome coverage and SNVs were characterized. To discern the influence of these quality parameters on critical surveillance metrics, consensus genomes were subjected to PANGO lineage assignment and phylogenetic clustering.

#### Characterization of consensus genome quality

Genome coverage was calculated as the percentage of non-missing (N) alleles in a consensus genome relative to the SARS-CoV-2 reference genome (Wuhan-Hu-1; GenBank MN908947.3). Consensus genome positions where the observed alleles matched expected alleles were characterised as 'concordant', 'discordant' if the consensus allele did not match the expected allele, 'missing' if there was an N or gap at an expected SNV position of the SNV, or 'ambiguous' if any position contained an IUPAC ambiguity code.

#### Lineage assignment

**Fig. 1.**
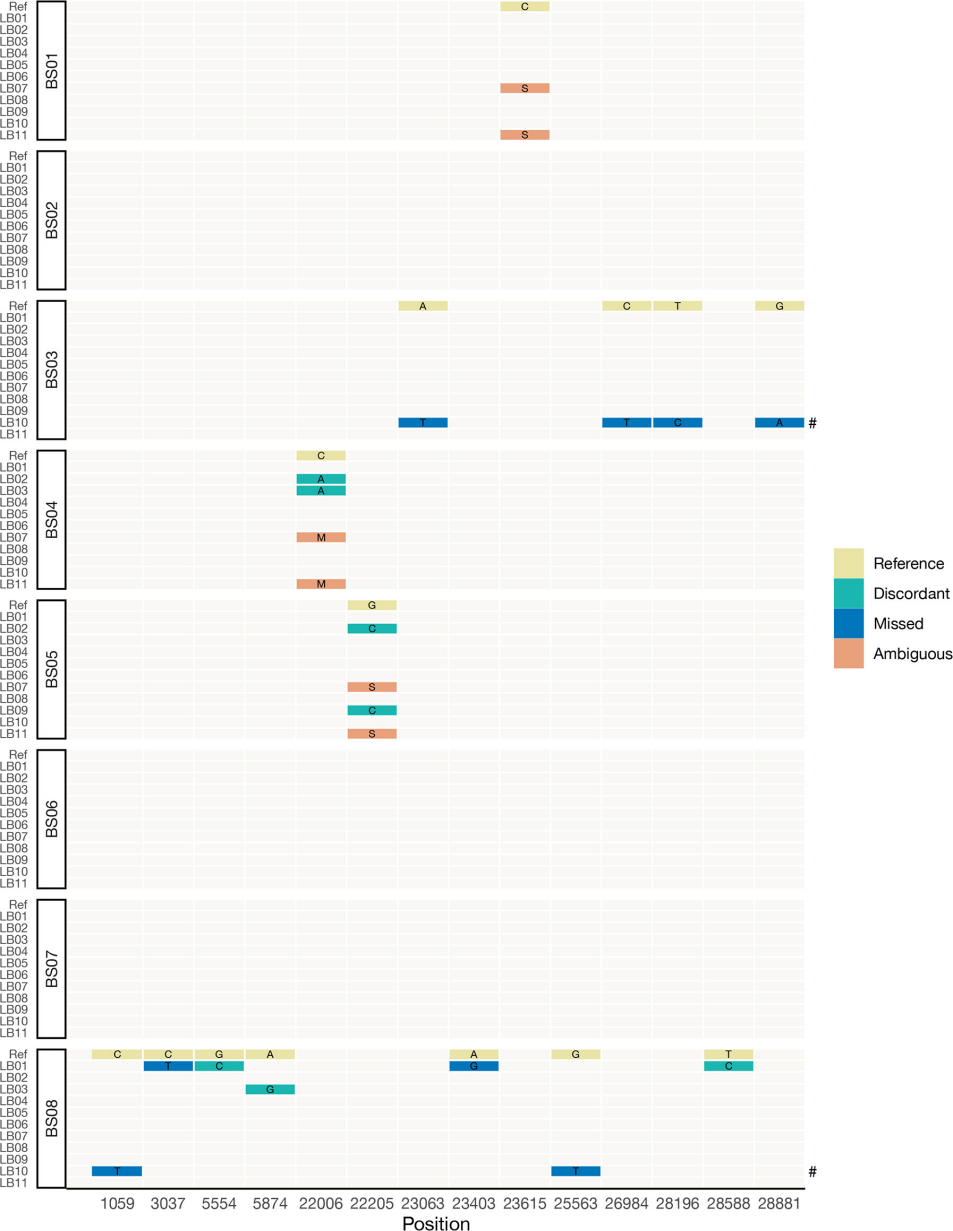
Discordant SNVs observed in participant consensus genomes submitted. Reference indicates the expected allele of the sample isolate reference sequence. Discordant SNVs are those where the observed participant allele differs from the expected reference allele. Missed SNVs are a subset of discordant SNVs in which a participant called an allele different from the expected SNV. Ambiguous SNVs are another subset of discordant SNVs in which the participant called an IUPAC ambiguity code where a standard base (ATGC) was expected (S=C or G; M=A or C). According to the criteria used in the PTP, the absence of C241T was considered concordant, and thus omitted from this figure if present. No annotation implies concordance. ^#^LB10 had additional discordant SNVs for samples BS03 (*N*=51) and BS08 (*N*=33) that are not listed here.

The consensus genomes submitted in FASTA format were used to obtain genome coverage and PANGO lineages. Lineages were assigned to the samples using Pangolin v.2.3.3 with pangoLEARN 2021-21-02, versions available at the time of the PTP. Discordant, missed and ambiguous SNVs relative to the expected SNVs defined by the PTP were identified using Nextclade v.2.8.0 (Fig. 1).

#### Phylogenetic clustering

A phylogenetic tree was constructed using RAxML-NG v.1.1 with a GTR+G4 model, starting trees from FastTree v.2.1.11 with 1000 bootstraps, and alignment column weight using gotree goalign compress v.0.4.2 [14]. BS03 and BS08 from LB10 were omitted from the final input alignment due to disproportionately long branches that negatively affected tree readability.

### Bioinformatic investigation of sequencing reads

PTP questionnaire responses lacked the necessary detail to accurately reconstruct individual bioinformatic methodologies. As a result, we adopted a standardized workflow for both Illumina and Nanopore data, investigating base-pair attributes at discordant sites and among amplicons, examining both trimmed and untrimmed reads.

#### Generating read pileups

FASTQ reads were aligned to the SARS-CoV-2 reference using minimap2 v. 2.24-r1122 with the options -axe map-ont for Nanopore or -axe sr for Illumina reads. Relevant amplicon primers (Table S1) were trimmed from the aligned reads via Samtools ampliconclip v.1.16.1, employing options –both ends –strand –soft-clip -u.

#### Obtaining base-pair attributes

Base-pair attributes of the total aligned reads were quantified using bam-readcount v.1.0.1 with default settings and subsequently interpreted using the parse_brc.py script [15]. Trimmed reads were processed similarly with option -b 20 to discard low-quality reads. The read depth for each amplicon was determined using mosdepth v.0.3.3, utilizing the corresponding amplicon insert regions as the input for the bam file.

### Investigating iSNVs in primary isolates

Investigation of base-pair attributes identified heterozygous sites consistently present in three specimens across all participant samples. To verify the presence of heterozygous alleles in the primary isolates, which are likely iSNVs, we analysed their sequencing reads. These reads were obtained from amplicon sequencing using ARTIC V3 primers prior to the PTP and were sequenced on an Illumina iSeq. Base-pair attributes were obtained as described earlier.

## Results

### Summary of reported methodologies

Participant workflows varied for laboratory and bioinformatic methodologies (Tables S1 and S2). Out of 11 participants, nine performed multiplex amplicon sequencing, with seven using the ARTIC V3 primer scheme [16, 17], another using a modified ARTIC V3 primer scheme to create 1 kbp amplicons, and one using the 'Midnight' scheme [4, 18]. Two participants used a long-pooled amplicon approach, 'JSE', with primers that produce 2.5 kbp amplicons [19, 20]. Most of the participants used an Illumina sequencing platform (*N*=8/11), with 6/8 using ARTIC V3 primers. All participants using Nanopore technology used the artic-ncov2019 pipeline (https://github.com/artic-network/artic-ncov2019). Amongst Illumina users, four used an in-house analysis pipeline, two used a mix of command line and commercial software, and two used solely commercial software (CLC Genomics Workbench).

### Characteristics of consensus genome quality

#### Genome coverage and contamination

Mean genome coverage for all consensus sequences submitted of SARS-CoV-2 positive samples was 95.98 %. Mean coverage of BS08 was lower overall (93.75 %) due to a high cycle threshold value (Ct=27.48) [13]. LB05 and LB09 had consistent coverage <99 %, suggesting dropout of the amplicon(s). While most participants detected few to no SARS-CoV-2 reads in the negative control (BS06), LB03 and LB05 recovered 11.0 and 4.5 % of the genome, respectively, and LB10 recovered 79.2 % (Fig. S1, available in the online version of this article).

#### Discordant SNVs

Out of the 11 participants, only four (LB04-6,8) submitted genomes that were 100 % concordant. Notably, this group comprised all Nanopore users (LB04-6), each of whom utilized distinct primer schemes (JSE, ARTIC V3 and Midnight) (Fig. 1). Two participants (LB07,11) were excluded from 100 % concordance solely due to ambiguous bases. The remaining 5/11 participants had one or more discordant SNV. Except for LB10, discordant SNVs per participant ranged from one to four. In the consensus genomes submitted by LB10 for BS03 and BS08, there were 55 and 35 SNVs, respectively. Excluding ambiguity calls and LB10, participants generated 100 % concordant genomes for BS01-3 and BS07.

### Impact on key surveillance metrics

#### Lineage assignment

**Fig. 2.**
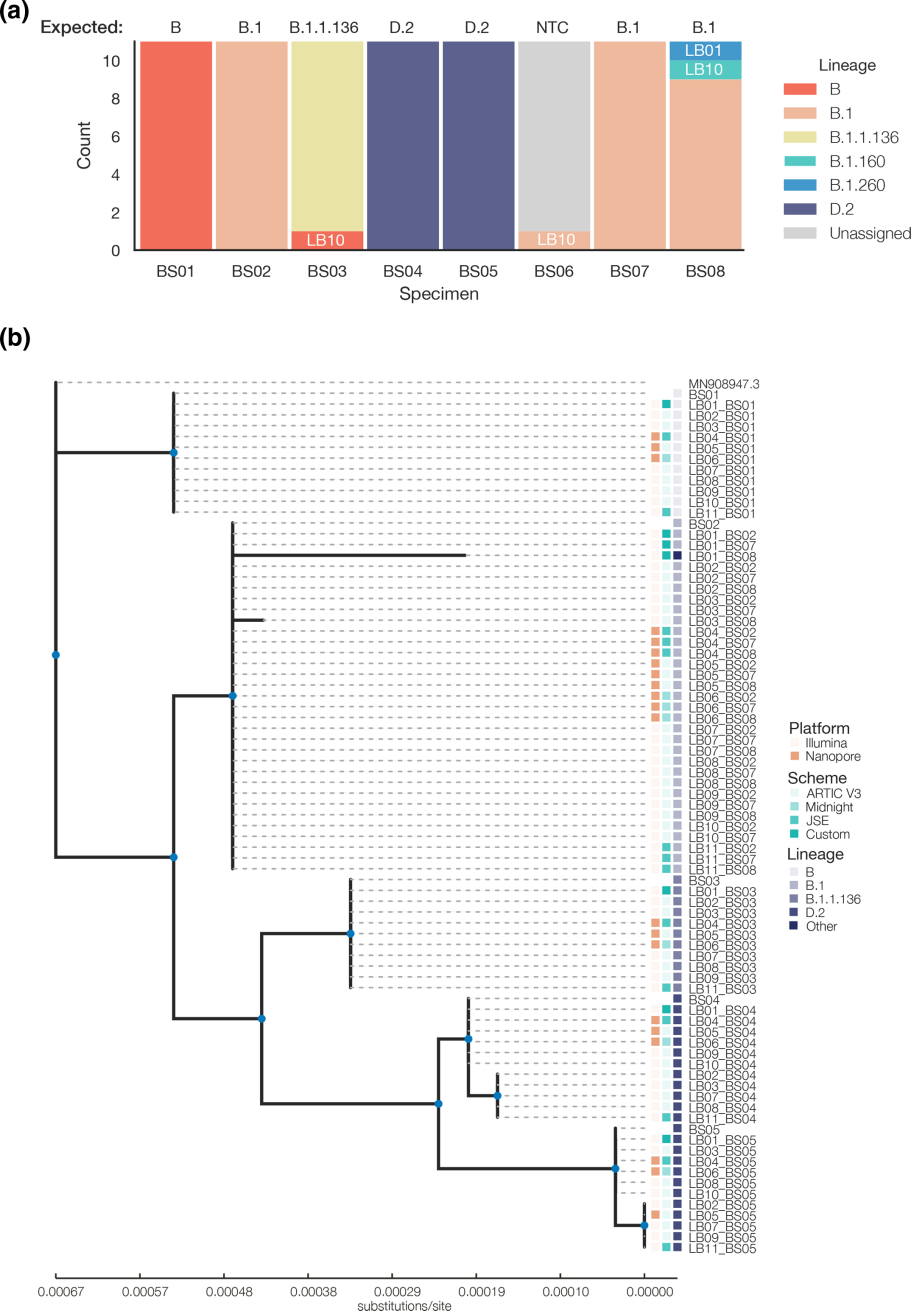
Key surveillance metrics derived from consensus genomes submitted. (**a**) Pangolin lineage assignments from consensus genomes submitted. Participant ID annotated where discordant. (**b**) Maximum-likelihood tree of consensus genomes submitted, excludes genomes<50 % coverage; and BS03 and BS08 for LB10. Nodes with teal markers indicate branch support >80 %. The heatmap denotes the sequencing platform, primer scheme, and PANGO lineage called for each tip. Phylogenetic tree visualized with toytree v1.0 (https://github.com/eaton-lab/toytree).

The PANGO lineage assignments generally accommodated missing data and false SNVs, with 95 % of consensus genomes submitted correctly designated at the highest taxonomic level (Fig. 2a). Because PANGOLIN classification is hierarchical, genomes with missing data can be expected to have parent lineage assignments, such as BS03 from LB10 [21]. BS08 samples from LB01 and LB10 might have been designated as a higher, incorrect lineage due to a combination of low genome coverage and false SNVs.

#### Phylogenetic clustering

In general, the consensus genomes submitted were placed in their expected phylogenetic clades (Fig. 2b). BS03 and BS08 from LB10 were excluded from the input alignment due to the presence of a high number of false mutations, leading to disproportionately long branches. Given the clonal nature of the PTP specimens, this introduces interpretative challenges in the tree. Out of the remaining 75 genomes, 84 % correctly fell within their expected clade. Subclades formed with low bootstrap support in BS04 and BS05, which included specimens with ambiguity codes, those that had a majority allele different from the isolate reference, and one specimen that was concordant with the basal clade. BS08 samples from LB01 and LB03 formed longer branches within the expected clade, with the former having the longest branch due to lower genome coverage (63.0 % vs. 96.7 %) and more discordant SNVs (*N*=4 vs. *N*=2).

### Bioinformatic investigation of sequencing reads

#### Amplicon dropouts

Per-amplicon read depth revealed LB05 had two or more amplicon dropouts across all samples, generally in amplicons 64 and 70. Similarly, LB09 failed to amplify amplicons 1 and 98 in all samples.

#### Base-pair attributes at heterozygous sites

**Fig. 3.**
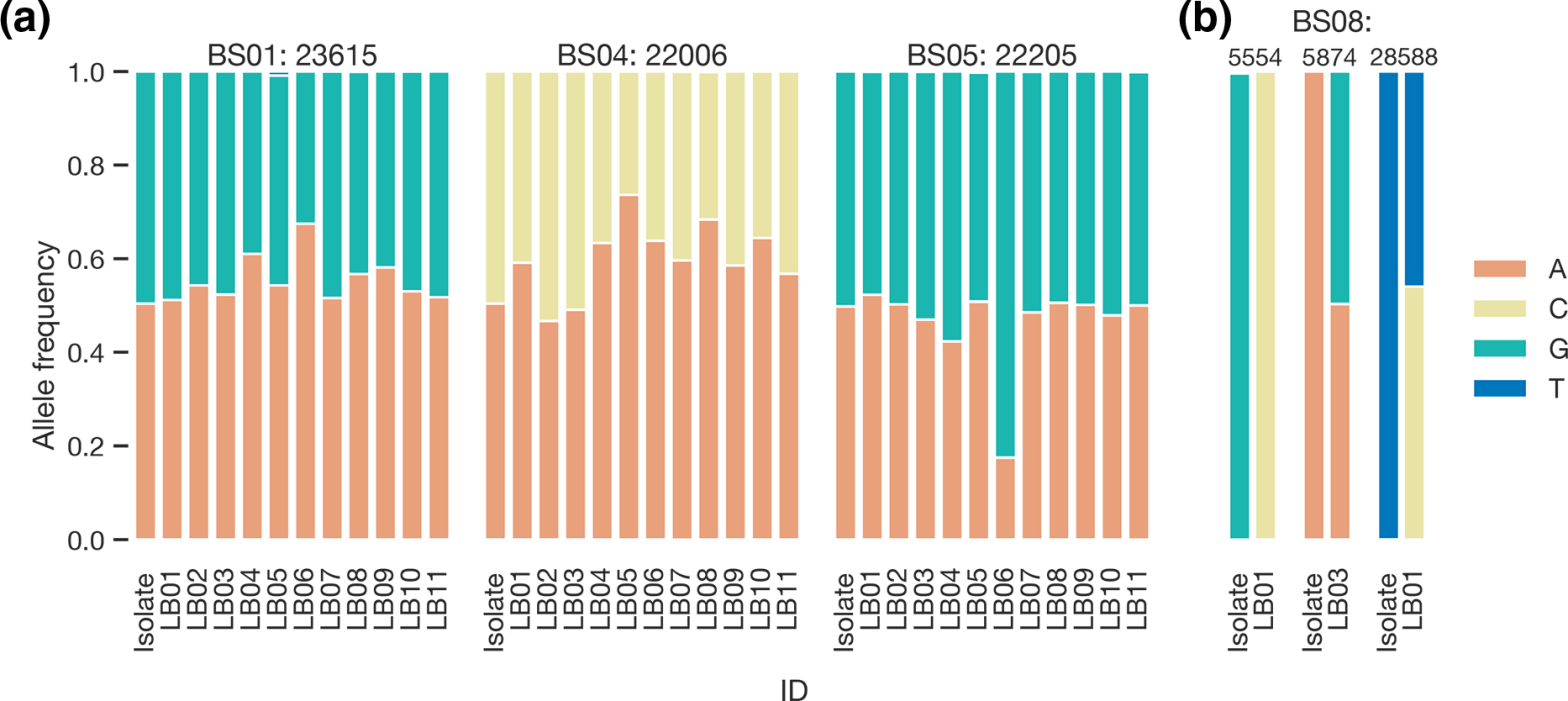
Allele distribution at discordant positions. (**a**) Allele frequencies at positions containing ambiguity in consensus genomes submitted to the PTP and the primary isolate used to create contrived specimen. (**b**) Allele frequencies at remaining discordant positions in both participant and primary isolate reads.

Inspection of read pileups at positions associated with ambiguous bases (BS01 : 23 615, BS04 : 22 006, BS05 : 22205) found that all participants had sequenced isolates with allele heterogeneity (Fig. 3a). Therefore, the allele called at the position depended upon (a) the allele mixture sequenced; (b) the variant calling threshold; and (c) whether ambiguity codes were used in variant calling. Taking this into account, six participants had 100 % concordant genomes, with an additional two having appropriate ambiguity codes. Examination of the alternate allele frequencies (ALT_FREQ) in the primary isolates at the previously specified loci reveals frequencies approximating 0.5 (Fig. S2). This pattern aligns with established observations of intrahost variation and cell-culture-adapted mutations seen in SARS-CoV-2 and various other viral isolates [21]. The majority of the remaining alternate alleles cluster around values of zero and one.

#### Base-pair attributes at discordant sites

In the high-Cτ sample BS08, LB01 and LB03 produced SNVs that were either discordant or missing. At nucleotide (nt) positions 25 588 and 5574 in LB01 and LB03, respectively, we observed an allele mixture where the discordant allele is the major allele according to the variant calling threshold used by the participant. We investigated the allele frequency at identical sites in the remaining samples and found them homogeneous. Finally, at nt position 5455 in the sample of LB01, the discordant allele was present at a frequency of 1 (Fig. 3b). Given that this false SNV was present in 100 % of the reads and was not detected in the other matched specimens, it may be the product of chimeric PCR amplification [7].

#### Analysis of problematic SNVs in LB10

LB10 had the highest number of discordant SNVs, with 55 and 35 in BS03 and BS08, respectively. Given the high recovery of the SARS-CoV-2 genome in the negative control of LB10 and the high number of total reads, laboratory contamination was suspected. However, an inspection of the read pileup found a disproportionately high read depth among several regions and found that the concordant allele was the major allele at discordant positions in many instances.

In BS03, there was overamplification of amplicons 2, 3 and 4 with a mean read depth of 43 866, 302 975 and 47 687, respectively. The mean depth across all other amplicons was 33.6. In BS08, the mean amplicon depth was 18.2. However, the read depth in the 3′UTR was 5910.7. Inspection of the read pileup in this region revealed an abundance of poly-A reads. All LB10 samples had similar read mapping characteristics in the 3′UTR, possibly suggesting poly-A carryover from cDNA synthesis.

When investigating the distribution of alleles at discordant positions, in many cases, the discordant allele was present at a depth of 1 or 0 (LB10-BS03 : 78.2 %; LB10-BS08 : 91.4 %) (Fig. S3). Where the expected concordant allele was present in all or a majority of reads at a discordant site, the mean frequency was 0.95 (median: 1.0; IQR: 0.05) and 0.96 (median: 1.0; IQR: 0.0) in LB10-BS03 and LB10-BS08, respectively. In fewer cases (LB10-BS03 : 14.5 %; LB10-BS08 : 5.71 %), the discordant allele was present in greater frequency than the concordant allele where the discordant allele had a mean frequency of 0.79 (median: 0.81; IQR: 0.23) and 0.62 (median: 0.62; IQR: 0.12) in LB10-BS03 and LB10-BS08, respectively. These observations of higher discordant allele frequency, along with the high presence of SARS-CoV-2 reads in the negative control specimen, may suggest these sites are the result of contamination.

We could not determine this participant’s specific bioinformatic methods or parameters from their questionnaire responses to further investigate how the variants were being called (Table S2). However, we can infer that the minimum depth threshold for variant calling was below 10, a commonly accepted minimum criterion for Illumina amplicon sequence data [8].

## Discussion

Here, we report findings from an in-depth retrospective analysis of discordant SNVs using consensus genomes, raw reads and the methodology questionnaire from a national SARS-CoV-2 WGS PTP. Despite diverse laboratory and bioinformatic methods, the data yielded largely consistent genomes, with discrepancies often resolved using robust bioinformatic thresholds. These discrepancies minimally affected key surveillance metrics. Recognizing that consensus genomes are generally accurate, certain challenges like low read depth and non-uniform amplicon sequencing coverage arise. As laboratory and bioinformatic techniques have advanced throughout the pandemic, there is a pressing need for continual refinement of SARS-CoV-2 WGS quality standards, which was facilitated here by the WG’s collaborative efforts.

### Flexibility of amplicon sequencing analyses

We find that consensus genomes generated with bioinformatic parameters at or above the commonly accepted minimum thresholds were highly accurate. It should be noted that two participants (LB03 and LB10) generated consensus genomes using read depth <10, the commonly accepted minimum threshold, with mixed results. LB03 used a depth of 3, resulting in 100 % concordant genomes when considering ambiguity. Based on the questionnaire and data analysis, it is suspected that LB10 may have utilized a depth of 1 for variant calling. Consequently, six of the eight consensus genomes exhibited 100 % concordance, whereas two (BS03 and BS08) displayed in excess of 40 discordant SNVs. One concern regarding variant calling with low read depth is false SNVs due to contamination, as observed here for a small number of false SNVs in LB10-BS03 and LB10-BS08. This can be ameliorated by monitoring reads in a negative sequencing control or a consensus sequence control to determine if a read set should use a higher depth threshold or fail quality control. The latter approach is more bioinformatically challenging to extract and interpret allele frequencies.

All Nanopore users (LB04-6) generated 100 % concordant genomes despite each using different primer schemes and lower read quality compared to Illumina users. Unlike Illumina users who used custom analysis scripts or commercial software, all Nanopore users employed the artic-ncov2019 pipeline (https://github.com/artic-network/artic-ncov2019). The robustness of the pipeline is unsurprising as it was designed especially for amplicon sequencing with Nanopore. While we could not reconstruct the analyses from the methodology questionnaire, our findings nonetheless suggest that further coordination between Illumina users is needed to troubleshoot problematic thresholds or standardize an analysis workflow.

### Considerations for assessing amplicon sequencing data

For PHLs actively involved in WGS quality control, they typically possess greater familiarity with methods tailored for prokaryotic pathogens, where shotgun sequencing of isolates is routinely conducted [22–24]. The characteristics of amplicon sequencing differ in several ways in which technical bioinformatic analysis can support laboratory optimisation. First, PCR efficiency often results in non-uniform coverage across the SARS-CoV-2 genome. Although genome coverage is an important quality indicator for unbiased sequencing approaches, it can be misleading when a small number of amplicons are overamplified or poly-A is carried through the library preparation and sequenced, as observed here with LB10. Instead, monitoring the mean read depth per amplicon would help participants troubleshoot amplicon drop outs, a common issue with this method that requires optimization [25–27]. Second, the proximity of discordant SNVs to primers can indicate bioinformatic trimming errors. It has previously been observed that CLC Genomics Workbench (Qiagen, https://www.qiagenbioinformatics.com/products/clc-genomics-workbench, accessed 03 January 2023) has a lower sensitivity to trimming partial primers and calling SNVs in primer regions, compared to iVar [7]. Although we were unable to replicate the results of LB10 using CLC 22 [*Identify ARTIC V3 SARS-CoV-2 Low-frequency and Shared Variants (Illumina) Workflow*] we did observe that false SNVs occurred near primer sites, which could signal the need for bioinformatic troubleshooting by the participant.

### Recommendations for assessing heterozygous sites

Similar to Foster *et al*. [6] [6], we primarily observed interlaboratory differences between matched samples when iSNVs, or heterozygous sites, were present. As in the assessments performed in Foster *et al*. [6] [6], where it was concluded IUPAC ambiguity codes were beneficial for quality control, we conclude these may signal laboratory contamination. They may also indicate epidemiological importance where true iSNVs arise from host-to-host transmission or co-infection [28, 29]. A good practice could be to call two consensus genomes using the following: (1) a strict minimum variant frequency threshold with ambiguity codes for the primary purpose of quality monitoring (i.e. a consensus sequence control); and (2) a major allele threshold without ambiguity codes for downstream analysis.

### Living guidelines for quality assessment during pandemics

Since the PTP analysed here concluded in 2021, several laboratory and bioinformatic developments have occurred, reflecting the evolving nature of the emergent pandemic response. The ARTIC primer scheme was redesigned in terms of laboratory methods and commercial schemes were offered to account for increasing variation [30, 31]. From a bioinformatic perspective, AusTrakka increased its minimum genome coverage criteria from >50 % to >=90 % (ACGT bases), which was the basis for the internal quality criteria of several participants. Regardless of AusTrakka participation, we agree with the conclusions of Lau *et al*. [13] and recommend this criterion as a minimum given the shared characteristics of the discordant metrics observed here (LB01-BS08, LB10-BS03 and LB10-BS08) having genome coverage <80 % in addition to previous benchmarking studies [8, 21]. These developments support the need for living guidelines to continually review and update the quality standards for SARS-CoV-2 WGS.

### Value of the WG to Australian PHLs

A key advantage of the WG was the real-time collaboration and knowledge-sharing to form living guidelines by continually reviewing and updating the quality standards for SARS-CoV-2 WGS. One member noted that by comparing results across laboratories and methodologies, they could identify and rectify a spurious SNV impacting tree topology, which stemmed from a specific primer set. Another highlighted the utility of the Slack channel, finding it valuable for accessing bioinformatic tools shared by colleagues, thereby enriching their analytical processes. Amid the pressing challenges of pandemic surges in PHLs, this collaborative approach promoted bioinformatic equity via shared expertise and distributed tasks. Additionally, it offered a dynamic platform for discussing the continuously evolving understanding and demands associated with the virus.

### Limitations

Given this was a retrospective analysis, we must consider that the PTP was not designed for the study at hand. While it offered a dataset reflecting SARS-CoV-2 WGS methodologies across Australia, its design posed challenges for our comparative analysis. Issues included data entry mistakes and inconsistencies in the PTP questionnaire responses. The questionnaire aimed to gauge the ability of participants to accurately report and convey results, but our analysis highlights potential challenges PHLs faced during pandemic surges. This might also underscore varying PHL capabilities in recognizing critical bioinformatic traits. For future reference, multidisciplinary working groups might consider creating an intuitive bioinformatic analysis platform for participants to upload data and obtain technical feedback, like the metrics described here. These insights would increase bioinformatic analysis equity and provide PHLs with data-driven strategies to inform living guidelines, facilitating sequence data concordance as knowledge and methods evolve throughout a pandemic.

## Conclusions

Despite the variety of laboratory and bioinformatic methods used for SARS-CoV-2 WGS, the results were largely accurate. However, challenges such as low read depths and inconsistent amplicon sequencing coverage were observed, and variants at positions with read depths below ten require careful consideration. We recommend PHLs bioinformatically generate two consensus genomes for distinct purposes: (1) quality control with ambiguity codes and a strict frequency threshold; and (2) downstream analysis without ambiguity codes. We also suggest implementing the following assessment criteria: (1) per-amplicon read depth as an indicator of primer efficiency; (2) SNVs to consider intrahost variation; and (3) a minimum genome coverage of 90 %. In a genomics-forward era of public health pathogen surveillance, our study emphasizes the role of multidisciplinary working groups in providing technical analysis and feedback for sequence data concordance across PHLs. We also present additional assessment criteria to guide laboratories in identifying areas for improvement.

## Supplementary Data

Supplementary material 1

Supplementary material 2

## References

[1] Chen Z, Azman AS, Chen X, Zou J, Tian Y (2022). Global landscape of SARS-CoV-2 genomic surveillance and data sharing. Nat Genet.

[2] Lane CR, Sherry NL, Porter AF, Duchene S, Horan K (2021). Genomics-informed responses in the elimination of COVID-19 in Victoria, Australia: an observational, genomic epidemiological study. Lancet Public Health.

[3] (2020). Issues with SARS-Cov-2 sequencing data. Virological. https://virological.org/t/issues-with-sars-cov-2-sequencing-data/473.

[4] Freed NE, Vlková M, Faisal MB, Silander OK (2020). Rapid and inexpensive whole-genome sequencing of SARS-CoV-2 using 1200 bp tiled amplicons and Oxford Nanopore rapid barcoding. Biol Methods Protoc.

[5] Quick J, Grubaugh ND, Pullan ST, Claro IM, Smith AD (2017). Multiplex PCR method for MinION and Illumina sequencing of Zika and other virus genomes directly from clinical samples. Nat Protoc.

[6] Foster CSP, Stelzer-Braid S, Deveson IW, Bull RA, Yeang M (2022). Assessment of inter-laboratory differences in SARS-CoV-2 consensus genome assemblies between Public Health Laboratories in Australia. Viruses.

[7] Liu T, Chen Z, Chen W, Chen X, Hosseini M (2021). A benchmarking study of SARS-CoV-2 whole-genome sequencing protocols using COVID-19 patient samples. iScience.

[8] Xiaoli L, Hagey JV, Park DJ, Gulvik CA, Young EL (2022). Benchmark datasets 507 for SARS-CoV-2 surveillance bioinformatics. PeerJ.

[9] Rambaut A, Holmes EC, O’Toole Á, Hill V, McCrone JT (2020). A dynamic nomenclature proposal for SARS-CoV-2 lineages to assist genomic epidemiology. Nat Microbiol.

[10] Frampton D, Rampling T, Cross A, Bailey H, Heaney J (2021). Genomic characteristics and clinical effect of the emergent SARS-CoV-2 B.1.1.7 lineage in London, UK: a whole-genome sequencing and hospital-based cohort study. Lancet Infect Dis.

[11] Geoghegan JL, Ren X, Storey M, Hadfield J, Jelley L (2020). Genomic epidemiology reveals transmission patterns and dynamics of SARS-CoV-2 in Aotearoa New Zealand. Nat Commun.

[12] Hoang T, da Silva AG, Jennison AV, Williamson DA, Howden BP (2022). AusTrakka: fast-tracking nationalized genomics surveillance in response to the COVID-19 pandemic. Nat Commun.

[13] Lau KA, Horan K, Gonçalves da Silva A, Kaufer A, Theis T (2022). Proficiency testing for SARS-CoV-2 whole genome sequencing. Pathology.

[14] Lemoine F, Gascuel O (2021). Gotree/Goalign: toolkit and Go API to facilitate the development of phylogenetic workflows. NAR Genom Bioinform.

[15] Quick J (2019). nCoV-2019 sequencing protocol V3 (Locost). protocols.

[16] Tyson JR, James P, Stoddart D, Sparks N, Wickenhagen A (2020). Improvements to the ARTIC multiplex PCR method for SARS-CoV-2 genome sequencing using nanopore. bioRxiv.

[17] Freed N, Silander O (2021). SARS-CoV2 genome sequencing protocol (1200bp amplicon “midnight” primer set, using Nanopore Rapid kit). protocols.

[18] Eden J-S, Rockett R, Carter I, Rahman H, de Ligt J (2020). An emergent clade of SARS-CoV-2 linked to returned travellers from Iran. Virus Evol.

[19] Eden J-S, Sim E SARS-CoV-2 genome sequencing using long pooled amplicons on illumina platforms v1. protocols.

[20] O’Toole Á, Scher E, Underwood A, Jackson B, Hill V (2021). Assignment of epidemiological lineages in an emerging pandemic using the pangolin tool. Virus Evol.

[21] Aiewsakun P, Phumiphanjarphak W, Ludowyke N, Purwono PB, Manopwisedjaroen S (2023). Systematic exploration of SARS-CoV-2 adaptation to vero E6, vero E6/TMPRSS2, and calu-3 cells. Genome Biol Evol.

[22] Lau KA, Gonçalves da Silva A, Theis T, Gray J, Ballard SA (2021). Proficiency testing for bacterial whole genome sequencing in assuring the quality of microbiology diagnostics in clinical and public health laboratories. Pathology.

[23] Moran-Gilad J, Sintchenko V, Pedersen SK, Wolfgang WJ, Pettengill J (2015). Proficiency testing for bacterial whole genome sequencing: an end-user survey of current capabilities, requirements and priorities. BMC Infect Dis.

[24] Gargis AS, Kalman L, Lubin IM, Kraft CS (2016). Assuring the quality of next-generation sequencing in clinical microbiology and public health laboratories. J Clin Microbiol.

[25] Borcard L, Gempeler S, Terrazos Miani MA, Baumann C, Grädel C (2020). Investigating the extent of primer dropout in SARS-CoV-2 genome sequences during the early circulation of delta variants. FrontVirol.

[26] Lambisia AW, Mohammed KS, Makori TO, Ndwiga L, Mburu MW (2022). Optimization of the SARS-CoV-2 ARTIC network V4 primers and whole genome sequencing protocol. Front Med.

[27] Cotten M, Lule Bugembe D, Kaleebu P, Phan TMV (2021). Alternate primers for whole-genome SARS-CoV-2 sequencing. Virus Evol.

[28] Armero A, Berthet N, Avarre J-C (2021). Intra-host diversity of SARS-Cov-2 should not be neglected: case of the state of Victoria, Australia. Viruses.

[29] Lythgoe KA, Hall M, Ferretti L, de Cesare M, MacIntyre-Cockett G (2021). SARS-CoV-2 within-host diversity and transmission. Science.

[30] Davis JJ, Long SW, Christensen PA, Olsen RJ, Olson R (2021). Analysis of the ARTIC version 3 and version 4 SARS-CoV-2 primers and their impact on the detection of the G142D amino acid substitution in the spike protein. Microbiol Spectr.

[31] Bei Y, Pinet K, Vrtis KB, Borgaro JG, Sun L (2022). Overcoming variant mutation-related impacts on viral sequencing and detection methodologies. Front Med.

