# Supplementary material for: Bioinformatic investigation of discordant sequence data for SARS-CoV-2: insights for robust genomic analysis during pandemic surveillance
Source: Microb Genom. 2023 Nov 29;9(11):001146. doi: 10.1099/mgen.0.001146 (PMC10711311; doi:10.1099/mgen.0.001146)
Supplement: Supplementary material 1 [file mgen-9-1146-s001.pdf]

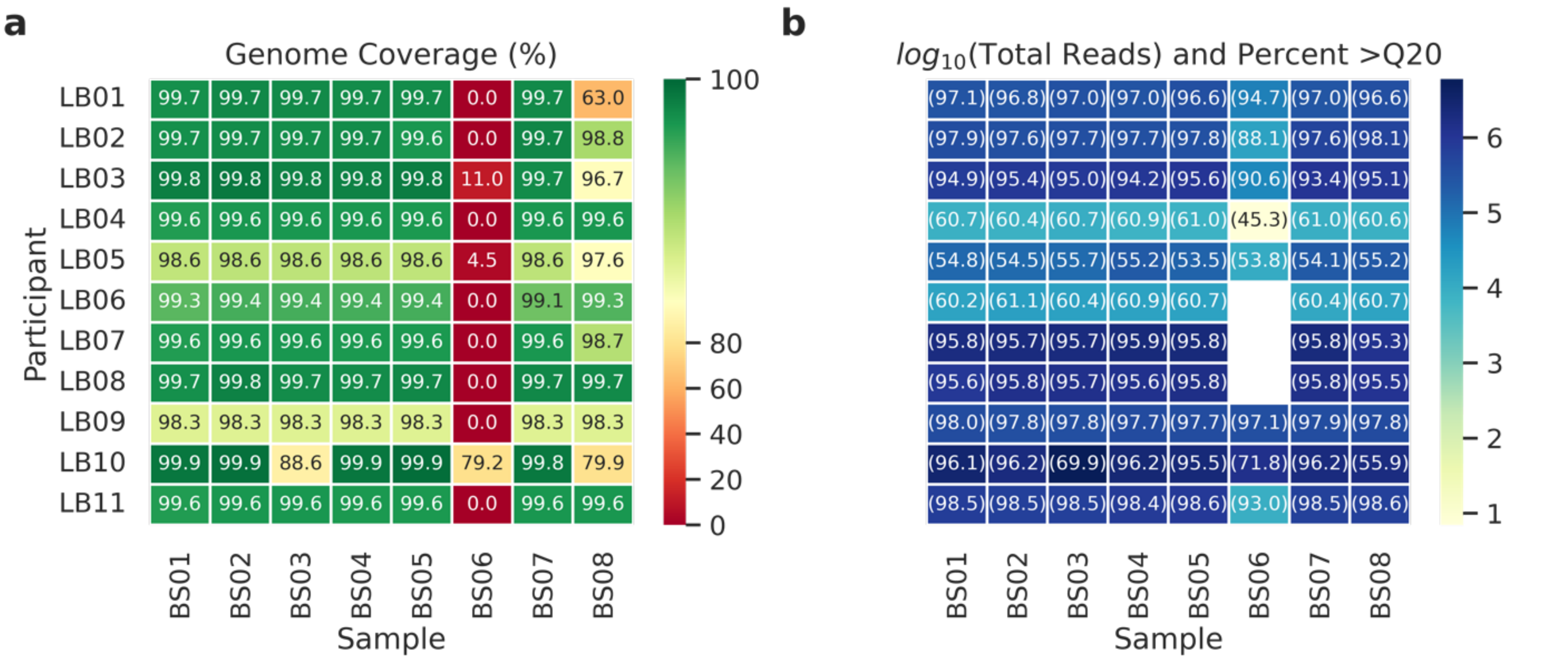

Supplementary Figure 1.

Characteristics of consensus genomes and reads submitted to the PTP. a) Genome coverage of consensus genomes submitted relative to the SARS-CoV-2 reference sequence (NC_045512.2). b) Number of reads per sample (log normalised), annotations indicate the percentage reads with quality score >Q20.

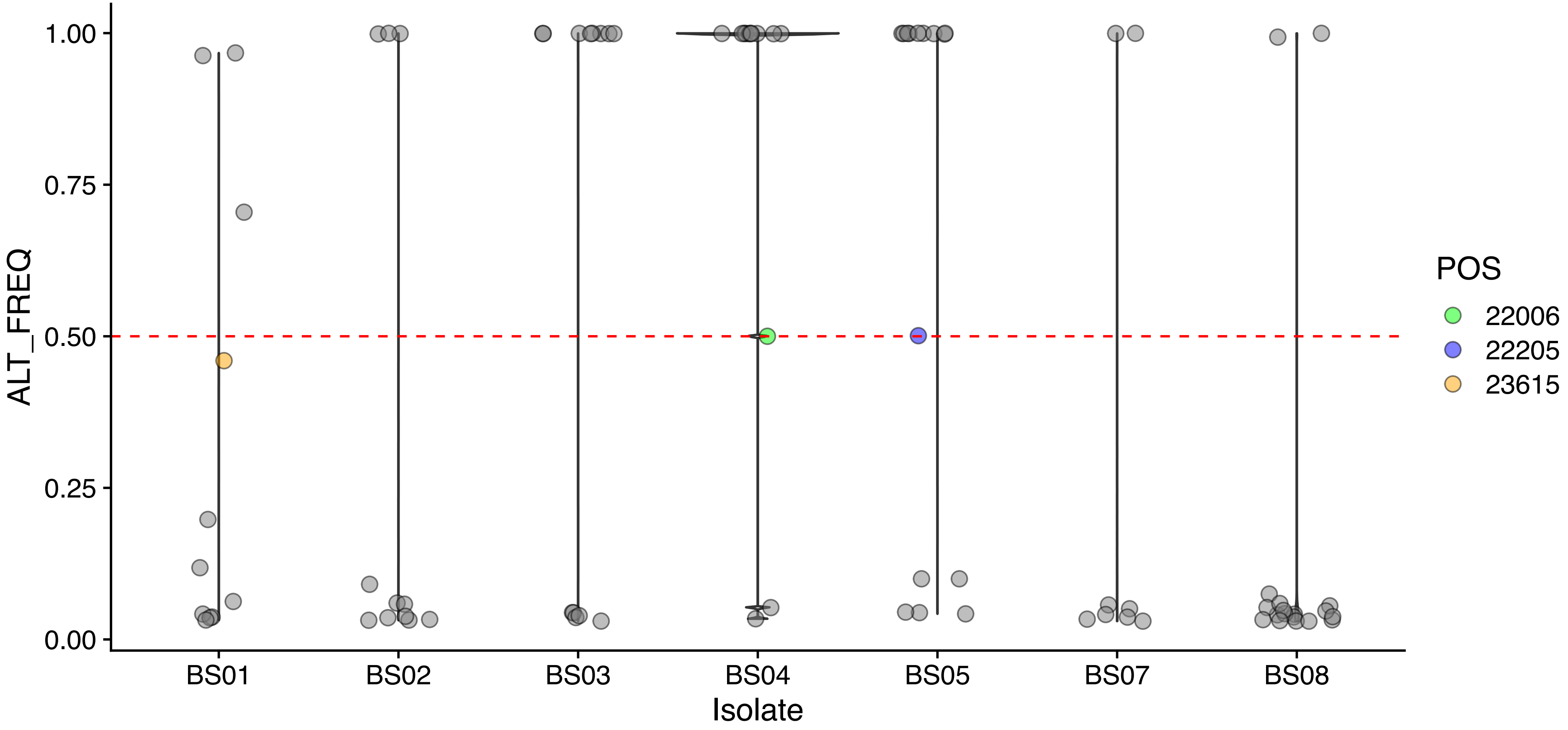

	ID	POS	REF	ALT	ALT_FREQ
1	BS01	23615	C	G	0.459740
2	BS04	22006	C	A	0.500000
3	BS05	22205	G	C	0.500956

Supplementary Figure 2.

Distribution of alternate allele frequencies across primary isolates. Violin plot illustrating the distribution of alternate allele frequencies for each isolate. Overlaid on the violin plots are points colored according to the presence of variant positions of notable heterozygouse sites observed in PTP samples. The red dashed line represents a frequency of 0.5, highlighting the positions where alleles are nearly equally represented. Below the plot is a tabular representation of variants with alternate allele frequencies between 0.4 and 0.6. Displayed columns from left to right: Isolate ID, variant position (POS), reference nucleotide (REF), alternate nucleotide (ALT), and alternate allele frequency (ALT_FREQ).

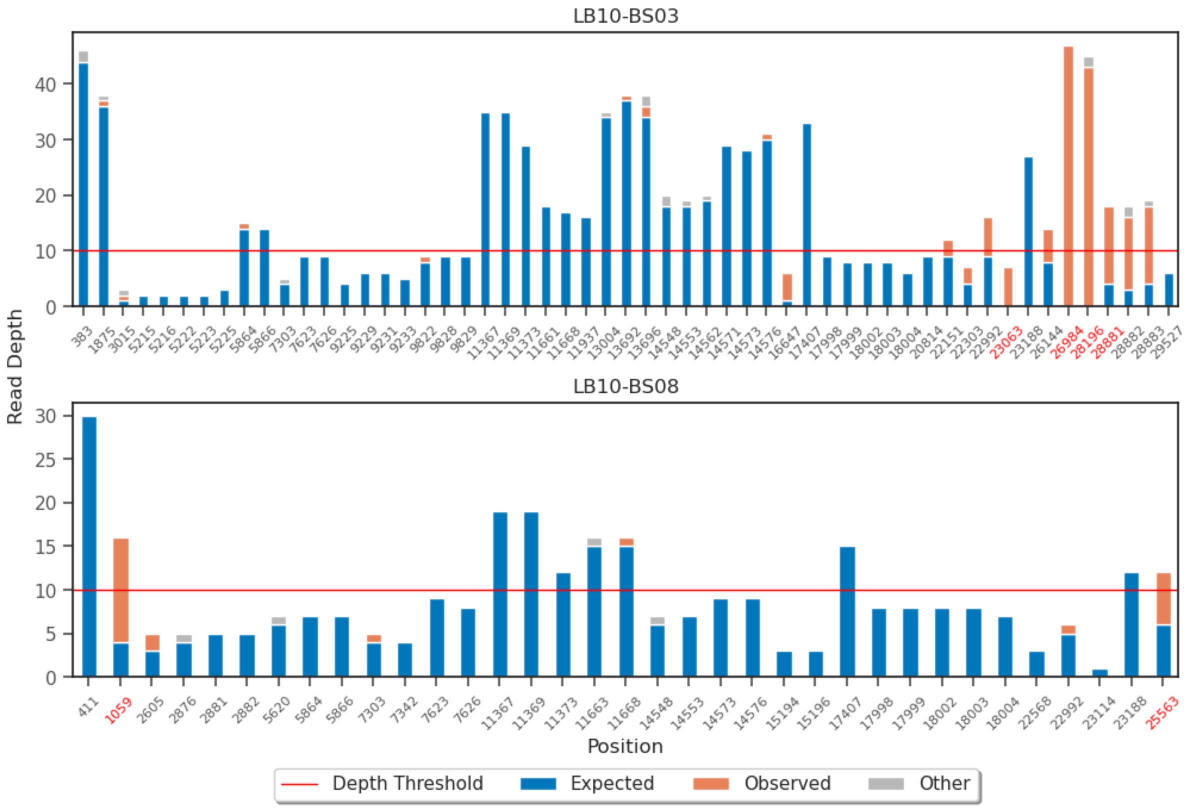

Supplementary Figure 3.

Allele frequencies at discordant positions from untrimmed reads of BS03 from LB10. The red horizontal line (Y=10) represents the generally accepted minimum read depth for variant calling for viral genomic surveillance. Nucleotide positions in red indicate the expected SNV sites.
